# Complete genome of an mpox clade 1b virus from Kenya

**DOI:** 10.1128/mra.00050-25

**Published:** 2025-04-11

**Authors:** Solomon K. Langat, Kimita Gathii, Konongoi Limbaso, Abdi Roba, Millicent Ndia, Beth Mutai, Genay Pilarowski, Melvin Ochieng, Bonventure Juma, Clayton Onyango, Albert Nyunja, Emmanuel Okunga, Victor Ofula, Paul Oluniyi, Edith Chepkorir, Joel Lutomiah, Amy Herman-Roloff, Naomi Lucchi, Hillary Limo, Daniel Langat, Samoel Khamadi, John Kiiru, Patrick Amoth, John Waitumbi, Elijah Songok

**Affiliations:** 1 Center for Virus Research, Kenya Medical Research Institute 118982, Nairobi, Kenya; 2 KEMRI/WRAIR-Africa, Basic Science Laboratory, Kisumu Field Station, Nairobi, Kenya; 3 National Public Health Laboratories, Nairobi, Kenya; 4 Chan Zuckerberg Biohub 578083, San Francisco, California, USA; 5 Diagnostic and Laboratory Systems Program (DLSP), Kenya Medical Research Institute 118982, Nairobi, Kenya; 6 Division of Global Health Protection, U.S. Centers for Disease Control and Prevention, Nairobi, Kenya; 7 Division of Disease Surveillance and Response, Ministry of Health, Nairobi, Kenya; 8 Public Health Emergency Operation Centre, Ministry of Health, Nairobi, Kenya; 9 Ministry of Health, Nairobi, Kenya; DOE Joint Genome Institute, Berkeley, California, USA

**Keywords:** mpox, clade Ib, Kenya, MPXV

## Abstract

We report the genome of a case of mpox detected in Kenya involving a truck driver with travel history to Uganda. Whole genome sequencing and phylogenetic analysis of the mpox virus (MPXV) showed that the genome clustered with clade Ib, which was recently identified in the Democratic Republic of Congo.

## ANNOUNCEMENT

Mpox is a zoonotic disease caused by monkeypox virus (MPXV) (Poxviridae family, *Orthopoxvirus* genus). It was first identified in 1958 among laboratory research primates (1, 2), with a human case detected in the Democratic Republic of Congo (DRC) in 1970 (3). There are two major clades of MPXV: clade I and clade II (4). Clade II includes subclades IIa and IIb, and in general has less severe symptoms compared with clade I (4). Clade IIb was involved in the 2022–2023 global outbreak, while a recently detected clade Ib lineage was reported to be the cause of an outbreak in the DRC (5, 6). Here, we report the genome of an index case of MPXV clade Ib virus in Kenya, which was detected in a middle-aged Kenyan male with a history of travel to Uganda.

**Fig 1:**
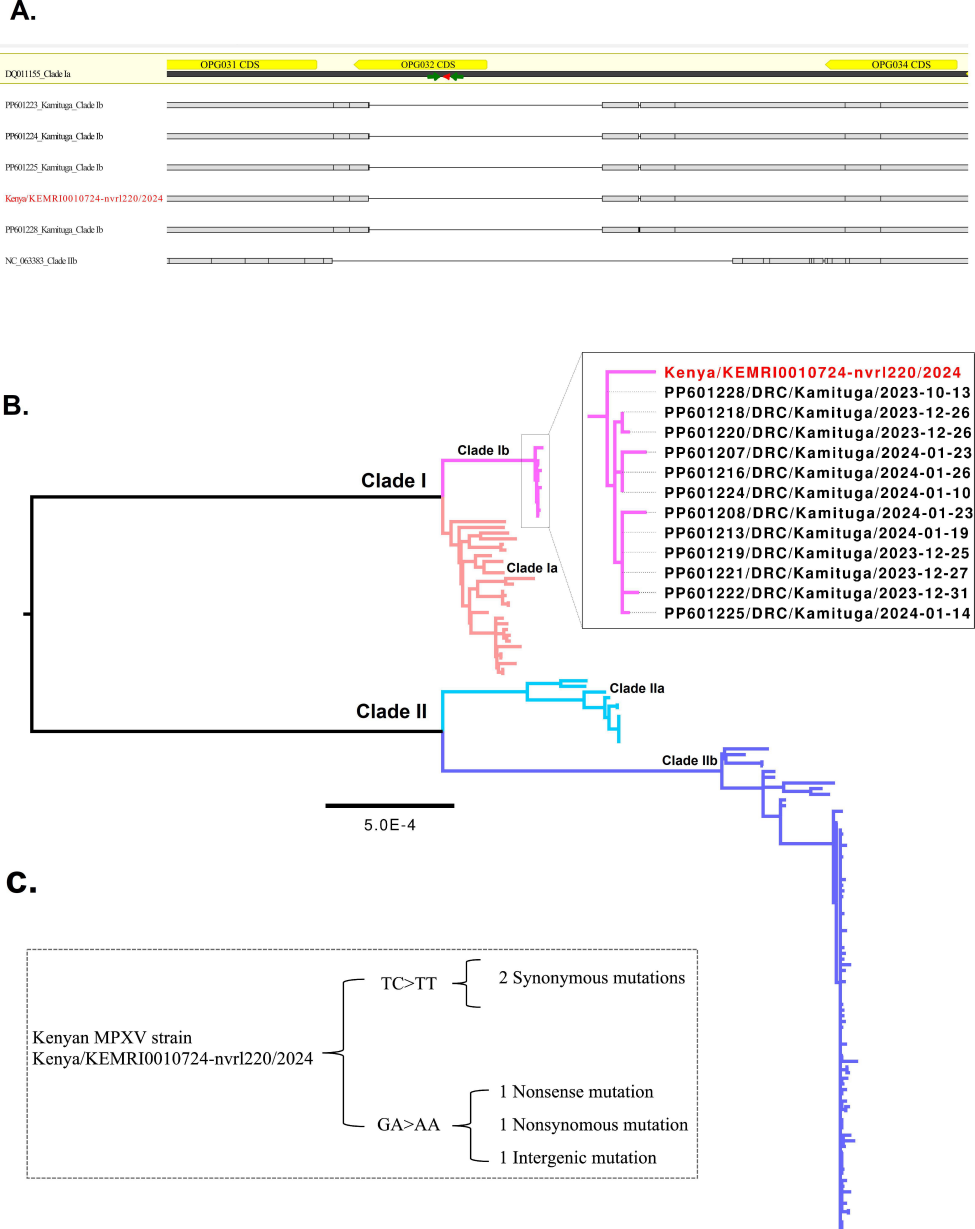
(A) The primer (green triangles) and probe (red triangles) binding region (targeting the C3L/CCP gene in MPXV). The gene is present in clade Ia strains but absent in clades Ib and IIb. (B) Maximum likelihood phylogeny based on complete genomes belonging to the two major clades of MPXV. The DRC outbreak clade has been expanded with the sequenced strain from Kenya indicated with a red tip label. (C) A summary of APOBEC3-like mutations carried by the Kenyan MPXV strain.

Skin lesion swabs were collected on 25 July 2024 and referred to the laboratory for testing. Genomic DNA was extracted using the MagNA Pure 24 Total NA Isolation kit (Roche, Basel, Switzerland) and tested for MPXV by qPCR using the F3L gene assay (7). It was confirmed positive for MPXV with a cycle-threshold (ct) of 21. However, clade I-specific Li et al. primers (8) did not amplify likely due to deletions within the C3L/CCP gene in clade 1b (Fig. 1A) (5).

MPXV sequencing utilized both long- and short-read approaches. For long read, DNA library was prepared using the native barcoding kit v14 (Oxford Nanopore Technologies, UK) and sequenced in a R10.4.1 flow cell on the PromethION for 48 h, with base calling performed simultaneously onboard using Guppy. For short reads, library preparation used the NEBNext Ultra II DNA Library Prep Kit for Illumina (NEB, UK) and Viral Surveillance Panel (Illumina, USA) as per manufacturer’s instructions. The libraries were sequenced in a 150-cycle paired-end configuration on the NextSeq2000 (Illumina, USA). Nanopore reads were checked for quality in Nanoplot (9), and Porechop v0.2.4 was used to remove adapters. The reads were assembled *de novo* using Flye (10), polished with Racon (11), and contigs assessed with Quast (12). Illumina reads were processed using Dragen Microbial Enrichment Plus v1.1.0 (Illumina, USA), which involved quality control, pathogen detection, and consensus sequence generation. Nanopore contigs were mapped against the Illumina genome using Minimap2 (13). MPXV consensus genome was further curated in Nextclade (14). All bioinformatic tools were run with default parameters unless otherwise specified. Our assembly yielded a genome of length 195,499 bp with a 33.1% G + C content and pairwise nucleotide identity score of 99.83% compared with the Zaire-96-I-16 strain (NC_003310.1), and 99.97% to the recent DRC isolate 24MPX0220V (PP601216.1).

Phylogenetic analysis and single nucleotide polymorphism (SNP) identification were performed using the squirrel bioinformatics pipeline (https://github.com/aineniamh/squirrel), with default parameters. The genome clustered with clade Ib genomes from the 2023/2024 DRC outbreak (Fig. 1B) (5). We identified six unique SNPs carried by the Kenyan strain. Five of these were APOBEC3-like mutations (Fig. 1C), which suggests host-mediated mutations during circulation among humans (15).

Successful sequencing and confirmation of MPXV clade Ib in Kenya points to continuing geographical expansion (16). This highlights the need for continued vigilance, including coordinated surveillance activities, increased in-country testing capability, and access to vaccines.

## Data Availability

The genome and associated data are available in GISAID (https://www.epicov.org/) under accession number EPI_ISL_19302262 and GenBank under accession number PQ178862. The raw sequence reads are available in the Sequence Read Archive (SRA) under BioProject PRJNA1147890 for both nanopore reads (SRR32413059) and Illumina reads (SRR30229922).
